# Supplementary material for: Multi-omics profiling reveals atypical sugar utilization and a key membrane composition regulator in Streptococcus pneumoniae
Source: Nat Commun. 2025 Nov 21;16:10429. doi: 10.1038/s41467-025-66611-0 (PMC12647141; doi:10.1038/s41467-025-66611-0)
Supplement: Supplementary file 1 — Supplementary information [file 41467_2025_66611_MOESM1_ESM.pdf]

Supplementary information

Multi-omics profiling reveals atypical sugar utilization and a key membrane composition regulator in *Streptococcus pneumoniae*

Vincent de Bakker, Xue Liu, Jonah Tang, Matthew Barbisan, Jonathon L. Baker, Jan-Willem Veening

Supplementary Table 1. **Growth conditions used in this study as defined by Aprianto *et al.* (2018)[1].** Sicard's defined medium was used as base medium for all conditions except C+Y, CSP and THY[1,2]. All cultures were kept in exponential growth phase at all times, with a maximum and final OD of 0.1.

	Niche-Mimicking Condition	T (°C)	pH[a]	Sugar (g/L)	Serum albumin (g/L)	CO_2 (%)	Experiment[b]	CRISPRi generations[c]
NMC	Nasopharynx	30	7	GlcNAc: 1.28	1	N.D.	T, P, F	7 (~12 h)
LMC	Lungs	37	7	GlcNAc: 0.64	3	5	T, P, F	7 (~7 h)
CSFMC	Cerebrospinal fluid	37	7.8	Glc: 0.45	0.45	5	T, P, F	7 (~9 h)
FEVER	Meningitis fever	40	7.8	Glc: 0.45	0.45	T/P: 5<br>F: N.D.	T, P, F	7 (~24 h)
C+Y		37	6.8	Glc: 1.69<br>Scr: 0.29	0.68	T/P: N.D.<br>F: 5	T, P, F	14 (~7 h)
CSP		37	6.8	Glc: 1.69<br>Scr: 0.29	0.68	5	T, P, F	14 (~7 h)
BMC	Blood	37	7.4	Glc: 0.9	67	5	F	14 (~9 h)
THY		37	7.5	Glc: 2.0	0	5	F	14 (~10 h)

[a] pH was calibrated at the start of the experiment.
[b] Column indicates which omics layers were measured.
[c] Column indicates for how many overall generations the CRISPRi pool was grown as estimated from the whole culture OD_{595}.
GlcNAc: N-acetylglucosamine, Glc: glucose, Scr: sucrose, T: transcriptome, P: proteome, F: fitnessome, N.D.: Not Determined.

Supplementary Table 2. **Bacterial strains that were used in this work.**

ID	Genotype	Source
VL1	Wild type *Streptococcus pneumoniae* D39V serotype 2	Slager *et al.* (2018)[3]
VL1998 / DCI23	D39V, *bgaA*::Plac-*dcas9sp* (tet), *prs1*::PF6-*lacI* (gen)	Liu *et al.* (2017)[4]
VL2210	D39V, *prs1*::PF6-*tetR* (gen)	Liu *et al.* (2021)[5]
VL4012	D39V, *bgaA*::Ptet (tet)	Lab collection
VL6009	D39V, *prs1*::PF6-*tetR* (gen), *bgaA*::Ptet-*nagA* (tet), Δ*nagA* (ery)	This study
VL5893	D39V, *prs1*::PF6-*tetR* (gen), *bgaA*::Ptet-*nagB* (tet), Δ*nagB* (ery)	This study
VL5305	D39V, Δ*nagA* (ery)	This study
VL5306	D39V, Δ*nagB* (ery)	This study
VL7271	Δ*manLMN* (kan)	This study
VL7272	Δ*nagA* (ery), Δ*manLMN* (kan)	This study
VL7273	Δ*nagB* (ery), Δ*manLMN* (kan)	This study
VL6008	D39V, *prs1*::PF6-*tetR* (gen), *bgaA*::Ptet-*spv_0647-comEB* (tet), Δ*spv_0647-comEB* (ery)	This study
VL6007	D39V, *prs1*::PF6-*tetR* (gen), *bgaA*::Ptet-*comEB* (tet), Δ*comEB* (ery)	This study
VL6033	D39V, *prs1*::PF6-*tetR* (gen), *bgaA*::Ptet-*spv_0647* (tet), Δ*spv_0647* (ery)	This study
VL6477	D39V, CEP::PF6-*tetR*-Ptet (spc)	Lab collection
VL6935	D39V, *prs1*::Plac-*fakB3*-PF6-*lacI* (gen)	Lab collection
VL7362	D39V, *prs1*::Plac-*fakB3*-PF6-*lacI* (gen), CEP::PF6-*tetR*-Ptet-*fabM* (spc), Δ*spv_0647* (ery)	This study
VL6297	Δ*spv_0647* (ery)	This study

Notes:

- Plac: IPTG-inducible promoter, PF6: constitutive promoter, Ptet: tetracycline-inducible promoter (PT5-3 variant)[6]
- tet: tetracycline resistance, gen: gentamycin resistance, ery: erythromycin resistance, kan: kanamycin resistance, spc: spectinomycin resistance

Supplementary Table 3. **Oligonucleotides that were used in this study to construct strains.**

ID	Sequence (5'-3')	RE*
OVL7886	caactggtttaccatgcacacc	
OVL7887	TCGACGTCTCGAAgagagaaagcagaagttagaga	Esp3I
OVL7888	TCGACGTCTCGtcTTATTTCCTCCCGTTAAATAATAGAT	Esp3I
OVL7889	TCGACGTCTCGtATGAACAAAAATATAAAATATTCTCAAAACT	Esp3I
OVL7890	TCGACGTCTCGCATaatgttaacctccttaaaagattga	Esp3I
OVL7891	gtattggaaccttggattgcagg	
OVL7892	ggcaaagatatcgacacgg	
OVL7893	CAGAGGTCTCGaaagcaatgttctattgaacgc	BsaI
OVL7894	CAGAGGTCTCGcttttTTATTTCCTCCCGTTAAATAATAGAT	BsaI
OVL7895	CAGAGGTCTCGagATGAACAAAAATATAAAATATTCTCAAAACT	BsaI
OVL7896	CAGAGGTCTCCATcttttcatcctccatttctgtc	BsaI
OVL7897	ggtccaaccagaatctgcttgg	
OVL8025	ggtcacctcttgtcaagaatgc	
OVL8026	GAGTCGTCTCGCATaacattttctttctacttgtcaca	Esp3I
OVL8027	GAGTCGTCTCCtATGAACAAAAATATAAAATATTCTCAAAACT	Esp3I
OVL8028	GAGTCGTCTCCTTTCCTCCCGTTAAATAATAGATAAC	Esp3I
OVL8029	GAGTCGTCTCGGAAATAAaaattatcaaaaataaatggtttagaaagatttttaaacc	Esp3I
OVL8030	ggacctgtcagcataatgatgc	
OVL8026	GAGTCGTCTCGCATaacattttctttctacttgtcaca	Esp3I
OVL8027	GAGTCGTCTCCtATGAACAAAAATATAAAATATTCTCAAAACT	Esp3I
OVL8882	GTAGCGTCTCGttctTTATTTCCTCCCGTTAAATAATAGAT	Esp3I
OVL8883	GTAGCGTCTCGagaaaaaggagaaaagagatgac	Esp3I
OVL8693	caagattgctgagccacctg	
OVL8884	CTCAGCTCTTCCctcttttctcctttttctctattttt	SapI
OVL8885	CTCAGCTCTTCCgagATGAACAAAAATATAAAATATTCTCAAAACT	SapI
OVL8886	CTCAGCTCTTCCttTTATTTCCTCCCGTTAAATAATAGAT	SapI
OVL8887	CTCAGCTCTTCCAaaattatcaaaaataaatggtttagaaagatt	SapI
OVL3830	CAACTCACATGAACTACATGATGAACCCAG	
OVL8498	GCGTCACGTCTCAATCTTTTGAATTCGCGGCCGC	Esp3I
OVL5352	GCGCTCAGCTCTTCAGATCTTTTGAATTCGCGGCCGC	SapI
OVL5019	GCGTCACGTCTCAACTAGTCAAGGTCGGCAATTC	Esp3I
OVL5353	GCGCTCAGCTCTTCAACTAGTCAAGGTCGGCAATTC	SapI
OVL5144	AACCTGCTGCTACTGCTGCTTGGCT	
OVL8499	GCGTCACGTCTCAAGATaggaggttaacattatgcctaac	Esp3I
OVL8500	GCGTCACGTCTCATAGTttatgcttgataacgttttacgc	Esp3I
OVL8501	GCGTCACGTCTCAAGATtggaggatgaaaagatgaaag	Esp3I
OVL8502	GCGTCACGTCTCATAGTttatttttcgagtaagctaagcgc	Esp3I
OVL8503	GCGTCAGCTCTTCAATCagaaagaaaatgttatgtctgaac	SapI
OVL8504	GCGTCAGCTCTTCAAGTctattttttaaaaaatggtaaacc	SapI
OVL8879	GCGTCACGTCTCAAGATaggagaaaagagatgactg	Esp3I
OVL8506	GCGTCACGTCTCATAGTttacattagatcagcctc	Esp3I
OVL8880	GCGTCAGCTCTTCAATCagaaagaaaatgttatg	SapI

OVL8881	GCGTCAGCTCTTCAAGTttacattagatcagc	SapI
OVL10267	Cgtcagcgttgcttggttc	
OVL10272	ccaaacacctcaacaagatgg	
OVL10746	GTCAcgtctcgCTGCtcgaagatttcagcttg	Esp3I
OVL10747	GTCAcgtctcgGCTTctagcaaaaaactggacg	Esp3I
OVL10748	GTCAcgtctcgAAGCtcaagcaactaaaaaggaaccagg	Esp3I
OVL10749	GTCAcgtctcgGCAGtattgtcattcctccttt	Esp3I
OVL9480	Ggagagattcaggtcaacattgaag	
OVL7825	ATGATTCTCAGACATCTGGGAATTAGC	
OVL10858	CGATcacctgccgtaTAACtcgagaaaaaaaaaccg	AarI
OVL10859	CGATcacctgccgtaGTTAttaatcaatttcatagcccatcag	AarI
OVL10860	CGATcacctgccgtaAATAatgacttggaagattattgctg	AarI
OVL10861	GCTAcacctgccgtaTATTtttcctccttatttatttag	AarI
OVL10753	Cattagccttcttatcatctcc	
OVL10754	Gttgaagtcagctaagctcg	
OVL10678	ATCGcgtctcgAATAatggaacacattatttatcagcttg	Esp3I
OVL10679	ATCGcgtctcgATCCttattttcctataaatttaggtcttcttc	Esp3I
OVL4212	GATCCGTCTCGTATTTTTCCTCCTTATTTATTTAGATCTACTCTA	Esp3I
OVL9724	GATCCGTCTCGGGATCCCTCCAGTAACTCG	Esp3I

Notes:

*RE: Restriction enzyme site introduced by PCR.

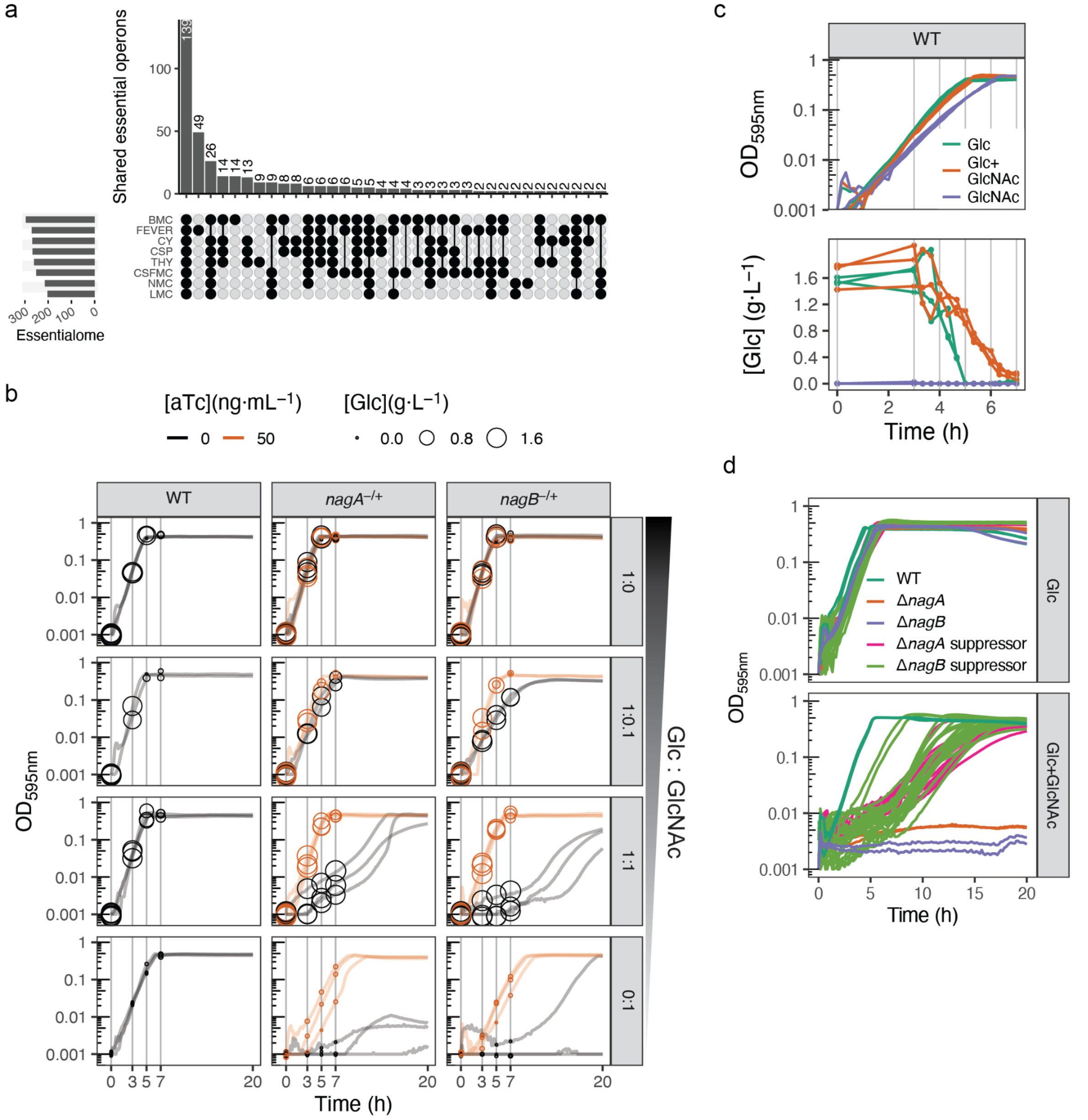

Supplementary Figure 1. **CRISPRi-seq profiles with focus on metabolism.** (**a**) General overlap of essentialomes between growth conditions: number of sgRNA targets classified as essential ($\log_2$FC<-1, P_{adj}<0.05). (**b**) Glucose concentrations in the medium during growth of WT strains and aTc-inducible ectopic complementation P_{tet}-*nagA* and P_{tet}-*nagB* strains in which the native genes are deleted, respectively (*nagA*$^{-/+}$, *nagB*$^{-/+}$). Glucose quantifications are superimposed on the corresponding sample growth curves for which they were measured. Media contained varying glucose:GlcNAc molar ratios, as indicated. Biological triplicates are shown. (**c**) Glucose concentrations in the medium, measured every 20 min, during growth of WT cells in glucose, GlcNAc, or an equimolar mix of the two. Biological triplicates are shown. (**d**) Growth curves of WT, *nagA* and *nagB* deletion mutants and all the suppressor isolates of the mutants, grown on glucose or an equimolar mix of glucose and GlcNAc. Technical duplicates are shown. Source data are provided in Supplementary Data 1 and as a Source Data file under Fig. 2a-b and SFig. 1d.

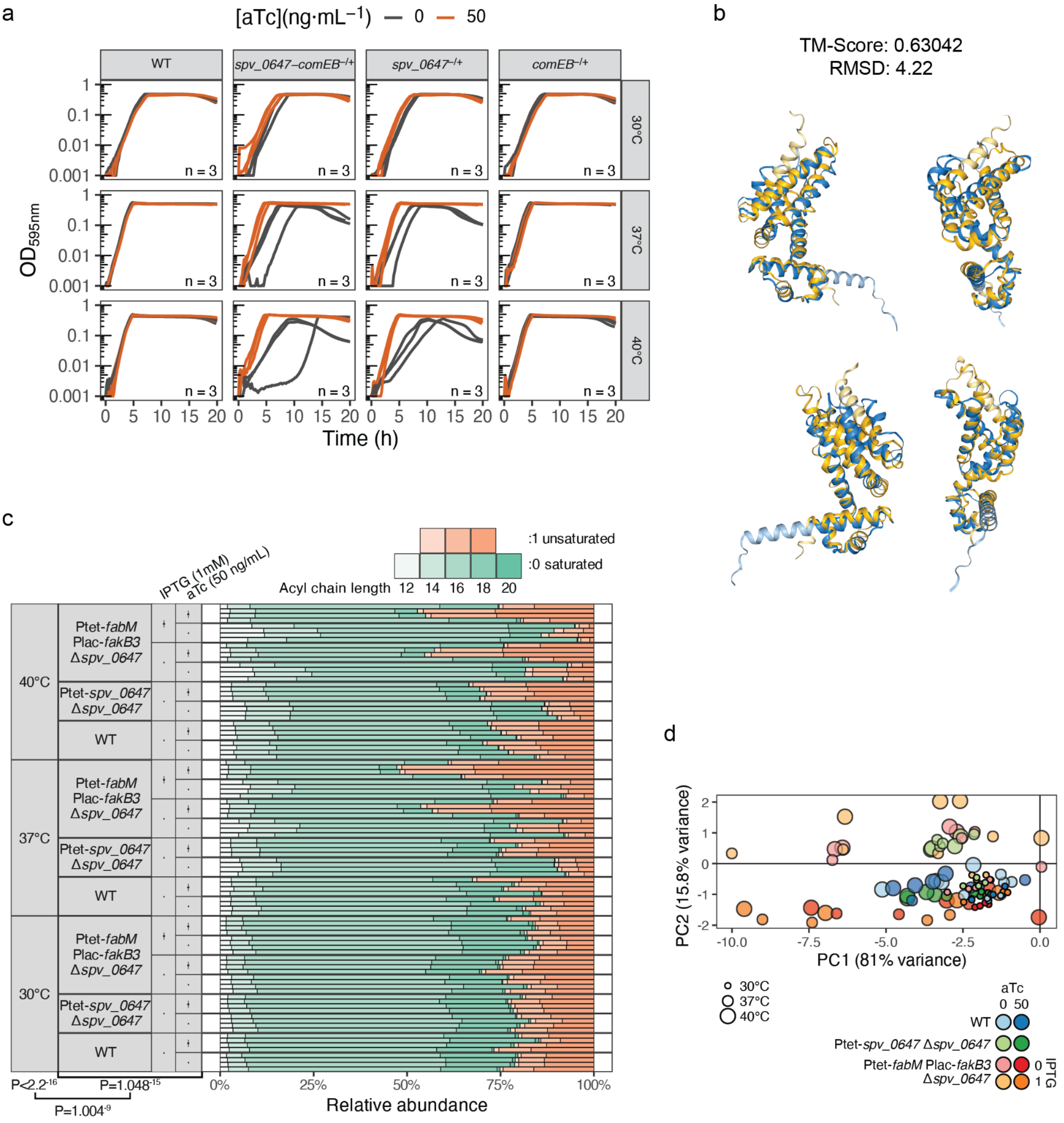

Supplementary Figure 2. ***spv_0647* (*fasR*) confers a fitness advantage during heat stress and influences fatty acid composition of the membrane.** (**a**) Growth curves of the WT strain, and *spv_0647*, *comEB* and the whole operon deletion mutants with aTc-inducible, ectopic complementation, grown at three different temperatures. Biological triplicates are shown. (**b**) Structural alignment of the FoldSeek top hit PDB ID 4MK6 (yellow) and SPV_0647 AlphaFold prediction (blue). (**c**) Full fatty acid membrane composition profiles of all temperature - strain combinations. P-values are derived from a compositional (mlm) ANOVA, performed using the R package compositions, based on an irl-transformation[7]. Biological quadruplicates are shown. (**d**) Principal component analysis of membrane composition profiles based on the Aitchison composition (acomp) transformation[7]. Source data are provided as a Source Data file and in Supplementary Data 4.

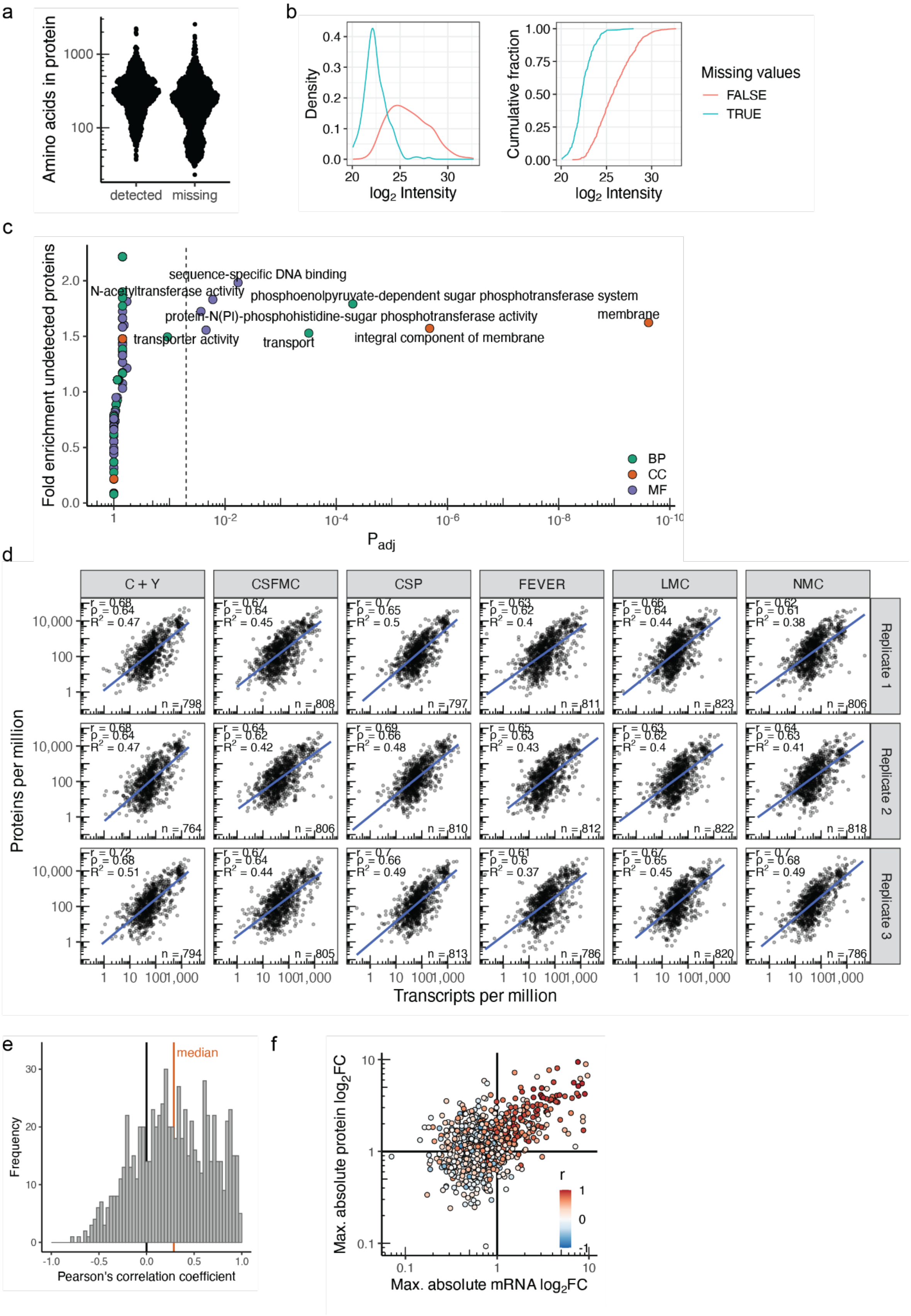

Supplementary Figure 3. **Transcriptome-proteome relationships across growth conditions.** (**a**) Shorter proteins were underrepresented in the data set. (**b**) Lowly abundant proteins were detected less frequently, presumably largely due to being close to the detection limit[8]. (**c**) GO term

enrichment analysis shows membrane proteins were overrepresented in the set of proteins left undetected. Fold enrichment is the gene ratio (fraction of undetected proteins in the GO set divided by the total of undetected proteins) divided by the background ratio (fraction of total proteins in the GO set divided by the total number of proteins). (**d**) Correlations between relative transcript and protein numbers per sample. The number of genes for which both levels were reliably quantified is displayed in each panel. (**e**) Distribution of correlation coefficients between transcript and protein levels across samples, per gene. Transcript numbers were normalized with the rlog transformation[9], and protein levels by vsn transformation following imputation of LFQ intensities[8]. (**f**) Maximum differential expression between any two growth conditions on the transcript and protein level. Correlations between transcript and protein levels across conditions as in panel (e). Source data are provided in Supplementary Data 5.

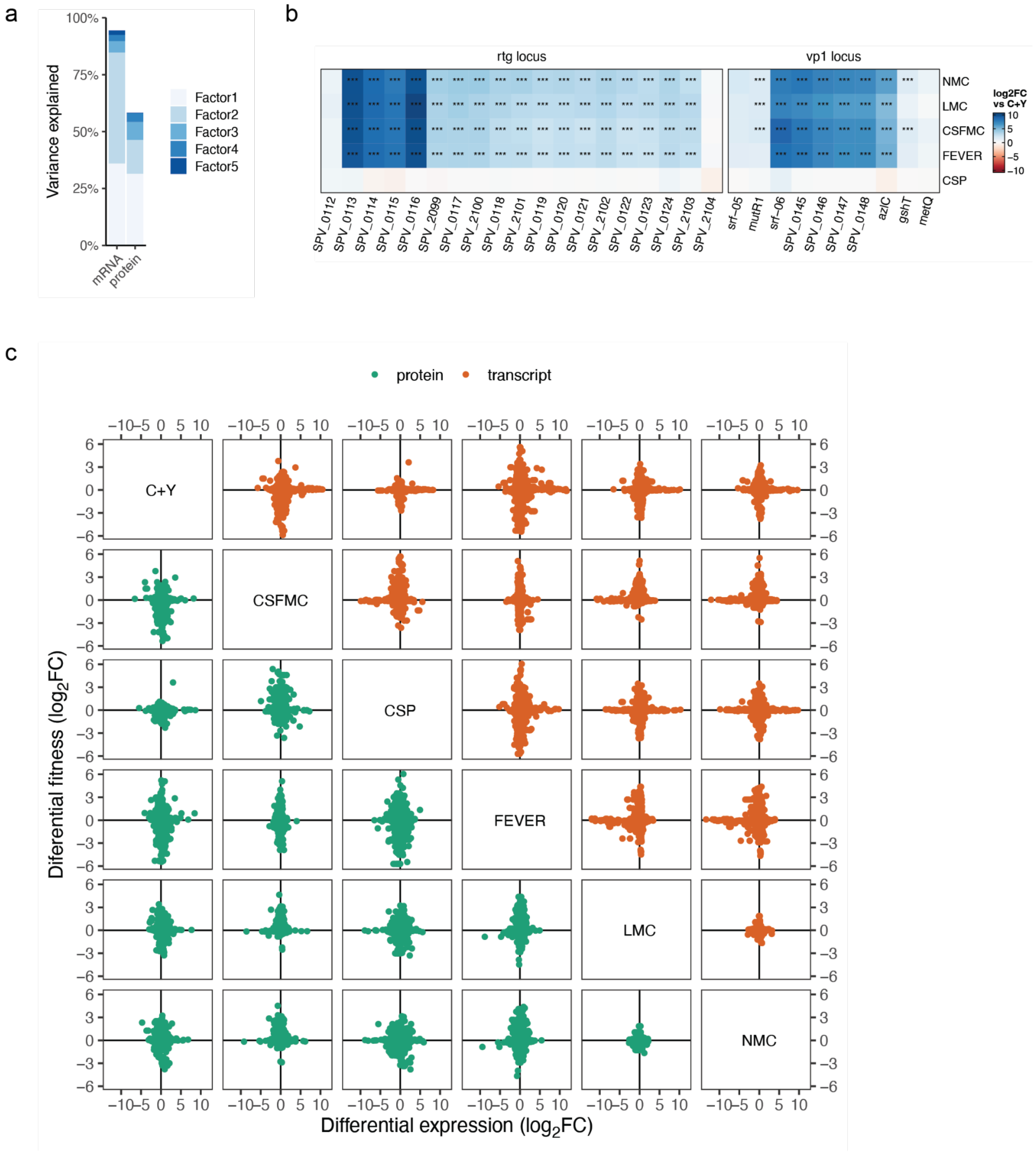

Supplementary Figure 4. **Integrated proteo-transcriptomic profiles.** (**a**) Variance explained per dimension in either dataset by Multi-Omics Factor Analysis (MOFA)[10]. (**b**) Differential expression of selected transcripts with high MOFA weights, compared to C+Y. Three asterisks indicates P_{adj}<0.001 for $|\Delta\log_2FC|>1$. Differential enrichment was tested using DESeq2 (negative binomial Generalized Linear Model with a Wald test) with two-tailed tests and P-values adjusted for False Discovery Rate[9]. (**c**) Comparison of differential expression (upper triangle: transcriptome, lower triangle: proteome) and fitness quantifications between each pair of growth conditions. Genes targeted by the same sgRNA (i.e., those sharing operons) were assigned the same fitness score. Source data are provided in Supplementary Data 1 and Supplementary Data 5.

References

1. Aprianto, R., Slager, J., Holsappel, S. & Veening, J.-W. High-resolution analysis of the pneumococcal transcriptome under a wide range of infection-relevant conditions. *Nucleic Acids Res.* **46** (2018) doi:10.1093/nar/gky750.
2. Sicard, A. M. A NEW SYNTHETIC MEDIUM FOR DIPLOCOCCUS PNEUMONIAE, AND ITS USE FOR THE STUDY OF RECIPROCAL TRANSFORMATIONS AT THE amiA LOCUS. *Genetics* **50**, 31-44 (1964) doi: 10.1093/genetics/50.1.31.
3. Slager, J., Aprianto, R. & Veening, J.-W. Deep genome annotation of the opportunistic human pathogen Streptococcus pneumoniae D39. *Nucleic Acids Res.* **46**, 9971–9989 (2018) doi:10.1093/nar/gky725.
4. Liu, X. et al. High-throughput CRISPRi phenotyping identifies new essential genes in Streptococcus pneumoniae. *Mol. Syst. Biol.* **13**, 931 (2017).
5. Liu, X. et al. Exploration of Bacterial Bottlenecks and Streptococcus pneumoniae Pathogenesis by CRISPRi-Seq. *Cell Host Microbe* **29**, 107-120.e6 (2021).
6. Sorg, R. A., Gallay, C., Van Maele, L., Sirard, J.-C. & Veening, J.-W. Synthetic gene-regulatory networks in the opportunistic human pathogen Streptococcus pneumoniae. *Proc. Natl. Acad. Sci.* **117**, 27608–27619 (2020).
7. Van Den Boogaart, K. G. & Tolosana-Delgado, R. Analyzing Compositional Data with R. (Springer Berlin Heidelberg, Berlin, Heidelberg, 2013). doi:10.1007/978-3-642-36809-7.
8. Zhang, X. et al. Proteome-wide identification of ubiquitin interactions using UbIA-MS. *Nat. Protoc.* **13**, 530–550 (2018).
9. Love, M. I., Huber, W. & Anders, S. Moderated estimation of fold change and dispersion for RNA-seq data with DESeq2. *Genome Biol.* **15**, 550 (2014).
10. Argelaguet, R. et al. Multi-Omics Factor Analysis—a framework for unsupervised integration of multi-omics data sets. *Mol. Syst. Biol.* **14**, e8124 (2018).
