# Supplementary material for: Multi-omics profiling reveals atypical sugar utilization and a key membrane composition regulator in Streptococcus pneumoniae
Source: Nat Commun. 2025 Nov 21;16:10429. doi: 10.1038/s41467-025-66611-0 (PMC12647141; doi:10.1038/s41467-025-66611-0)
Supplement: Supplementary file 7 — Description of Additional Supplementary Files [file 41467_2025_66611_MOESM7_ESM.pdf]

## Description of Additional Supplementary Files:

**Supplementary Data 1:** CRISPRi-seq data comprising sample metadata, raw and normalized sgRNA counts, and differential sgRNA enrichment results within and between growth conditions. Differential enrichment was tested using the R package DESeq2, fitting a negative binomial Generalized Linear Model and testing for significance with a two-tailed Wald test against an absolute log2 fold change threshold of 1. P-values were adjusted for multiple comparisons using the procedure of Benjamini and Hochberg.

**Supplementary Data 2:** RNA-seq data for wild-type and *Δspv_0647* mutant strains, comprising raw and normalized read counts, and differential expression results as tested by R package DESeq2, fitting a negative binomial Generalized Linear Model and testing for significance with a two-tailed Wald test against an absolute log2 fold change threshold of 1. P-values were adjusted for multiple comparisons using the procedure of Benjamini and Hochberg.

**Supplementary Data 3:** FoldSeek results generated through the online submission portal (https://search.foldseek.com) for UniProt accession number A0A0H2ZQ31 (SPV_0647), listing matches to the PDB100 data base.

**Supplementary Data 4:** GC-FAME results listing relative fatty acid levels per sample.

**Supplementary Data 5:** Proteo-transcriptomic data comprising raw and normalized quantifications for detected transcripts and proteins, and differential expression results. For transcripts, these were determined using the R package DESeq2, fitting a negative binomial Generalized Linear Model and testing for significance with a two-tailed Wald test against an absolute log2 fold change threshold of 1. For proteins, they were generated using R package DEP with label-free quantifications (LFQ values) as input, fitting a linear model and moderating t-statistic estimates by an empirical Bayes approach through the R package limma. P-values were adjusted for multiple comparisons by controlling the false discovery rate.
